# Post‐mortem oxygen isotope exchange within cultured diatom silica

**DOI:** 10.1002/rcm.7954

**Published:** 2017-09-14

**Authors:** Jonathan J. Tyler, Hilary J. Sloane, Rosalind E.M. Rickaby, Eileen J. Cox, Melanie J. Leng

**Affiliations:** ^1^ Department of Earth Sciences University of Adelaide Adelaide South Australia 5005 Australia; ^2^ NERC Isotope Geosciences Laboratory British Geological Survey Nottingham NG12 5GG UK; ^3^ Department of Earth Sciences The University of Oxford Oxford OX1 3AN UK; ^4^ The Natural History Museum London SW7 5BD UK; ^5^ Centre for Environmental Geochemistry University of Nottingham Nottingham NG7 2RD UK

## Abstract

**Rationale:**

Potential post‐mortem alteration to the oxygen isotope composition of biogenic silica is critical to the validity of palaeoclimate reconstructions based on oxygen isotope ratios (δ^18^O values) from sedimentary silica. We calculate the degree of oxygen isotope alteration within freshly cultured diatom biogenic silica in response to heating and storing in the laboratory.

**Methods:**

The experiments used freshly cultured diatom silica. Silica samples were either stored in water or dried at temperatures between 20 °C and 80 °C. The mass of affected oxygen and the associated silica‐water isotope fractionation during alteration were calculated by conducting parallel experiments using endmember waters with δ^18^O values of −6.3 to −5.9 ‰ and −36.3 to −35.0 ‰. Dehydroxylation and subsequent oxygen liberation were achieved by stepwise fluorination with BrF_5_. The ^18^O/^16^O ratios were measured using a ThermoFinnigan MAT 253 isotope ratio mass spectrometer.

**Results:**

Significant alterations in silica δ^18^O values were observed, most notably an increase in the δ^18^O values following drying at 40–80 °C. Storage in water for 7 days between 20 and 80 °C also led to significant alteration in δ^18^O values. Mass balance calculations suggest that the amount of affected oxygen is positively correlated with temperature. The estimated oxygen isotope fractionation during alteration is an inverse function of temperature, consistent with the extrapolation of models for high‐temperature silica‐water oxygen isotope fractionation.

**Conclusions:**

Routinely used preparatory methods may impart significant alterations to the δ^18^O values of biogenic silica, particularly when dealing with modern cultured or field‐collected material. The significance of such processes within natural aquatic environments is uncertain; however, there is potential that similar processes also affect sedimentary diatoms, with implications for the interpretation of biogenic silica‐hosted δ^18^O palaeoclimate records.

## 1 INTRODUCTION

The oxygen isotope composition of biogenic silica has become a widely used tool for reconstructing past climate and environmental change, particularly using diatom frustules extracted from lacustrine and marine sediments^1^, ^2^ and phytoliths extracted from plants and soils.^3^, ^4^, ^5^ In principle, the oxygen isotope composition of biogenic silica (δ^18^O_silica_, relative to Vienna Standard Mean Ocean Water (VSMOW)) reflects the temperature and oxygen isotope composition of water (δ^18^O_water_) in which the silica was precipitated, e.g. during diatom growth.^6^ However, since the pioneering analyses in the 1960s, the application of δ^18^O_silica_ values in palaeoclimatology has been beset by problems related to the secondary alteration of oxygen isotopes due to the presence of hydroxyl groups within the silica matrix.^6^, ^7^, ^8^, ^9^, ^10^, ^11^, ^12^, ^13^, ^14^

Amorphous biogenic silica (Si(OSi≡)_n_(OH)_4‐n_, where n ≤4) contains a significant mass of molecular water and hydroxylated silica (e.g. silanol, Si‐OH), in addition to tetrahedrally bonded Si‐O‐Si silica.^15^, ^16^, ^17^, ^18^, ^19^ The proportional concentration of silanols is reported as Q_*n*_, where Q represents a silicon atom surrounded by four oxygen atoms, while the suffix *n* gives the number of surrounding oxygen atoms (out of four) that are bonded to another silicon atom.^17^, ^18^, ^20^ Tetrahedrally bonded (Q_4_) silica is not known to undergo isotope alteration at temperatures <550 °C; however, hydroxyl‐bound oxygen within hydrated silica (Q_1–3_, herein referred to as Q_3_) readily exchanges with ambient water, such that a fraction of a given biogenic silica sample will undergo δ^18^O re‐equilibration under ambient conditions.^9^, ^14^ Methods have been developed to counter the presence of exchangeable Q_3_ silica during δ^18^O_silica_ analysis, most commonly by stepwise fluorination^21^, ^22^ or by controlled isotope exchange,^9^, ^23^, ^24^, ^25^ and those approaches have been demonstrated to produce reliable data between multiple laboratories.^26^ However, a number of researchers have observed differences between the δ^18^O_silica_ values of living and sedimentary diatoms, which suggest that the δ^18^O_silica_ value may not always faithfully record the signal acquired during diatom growth. For example, Schmidt et al,^10^ Moschen et al^27^ and Dodd et al^13^ all report significant increases in δ^18^O_silica_ values between living diatoms and those sampled from deep water traps or surface sediments. In some cases, these increases were also associated with the progressive loss of Q_3_ silica.^10^, ^27^ Similarly, a comparison of δ^18^O_silica_ values of living and surface sedimentary diatoms from Lochnagar, an upland lake in Scotland, revealed 4–6 ‰ δ^18^O_silica_ offsets between living and recently sedimented diatoms.^28^, ^29^ In the latter case, the sedimentary diatoms had lower δ^18^O_silica_ values than the living diatoms, with sedimentary values falling upon the line defined by a series of lake surface sediment δ^18^O_silica_ values across Europe.^30^ Partial dissolution of diatom frustules is one mechanism that may explain isotopic alteration during sedimentation; however, experimental studies suggest that the effects of dissolution upon diatom δ^18^O_silica_ values are small to negligible,^31^ contrasting with experimental evidence using phytoliths.^32^ Conversely, the process of silica condensation has been frequently proposed to impart significant effects upon δ^18^O_silica_ values.^10^, ^13^, ^14^, ^27^ Dodd et al^14^ demonstrated this effect by storing diatom silica in artificial sea water for 37 days, during which time the δ^18^O_silica_ value changed by approximately −5 ‰, alongside reductions in the relative mass of Si‐OH, both internally and upon the frustule surface. Dodd et al^14^ interpret their experimentally observed post‐mortem isotope exchange as a continuum towards complete isotopic re‐equilibration with secondary thermal and isotopic conditions. This interpretation contrasts with the widely held assumption (cited by Dodd et al^14^) that siloxane silica (Si‐O‐Si) requires a mineral phase change to undergo isotopic alteration.^33^ Furthermore, questions remain concerning the rate of oxygen isotopic exchange under different temperatures. Here we report an experiment based on simultaneous isotope ratio mass balance, designed to investigate secondary δ^18^O_silica_ alteration within freshly cultured diatom silica. Our results indicate that δ^18^O_silica_ values are sensitive to alteration on timescales of 1 week at high temperatures ≤80 °C, conditions which are commonplace in the storage and preparation of silica samples for isotope analysis.

## 2 EXPERIMENTAL

In order to test the isotope stability of biogenic silica under heating and drying conditions, a series of experiments was conducted using freshly cultured diatom silica. These experiments provide the basis for oxygen isotope mass balance calculations of the fractional mass of affected oxygen and the associated isotopic fractionation at temperatures relevant to natural sedimentation and laboratory pre‐treatment.

### 2.1 Experimental setup

Four batches of cultured diatom were used: three batches of Stephanodiscus hantzschii and one of Cyclotella meneghiniana. These species were chosen according to availability (via the Culture Collection of Algae and Protozoa, Scottish Association of Marine Science, Oban, UK; CCAP), their ease of culturing and their contrasting silica/biovolume ratios. Although both species are centric diatoms of similar habitat and morphological features, Stephanodiscus hantzschii has low silica mass/biovolume which contrasts with the high silica/biovolume of Cyclotella meneghiniana.^34^ Given the differences in silica/biovolume, it is reasonable to assume that frustules of these two diatom taxa also differ in their surface area/mass ratio, which in turn has been demonstrated to influence silica dissolution rate.^35^, ^36^ We therefore expected the denser frustule of Cyclotella meneghiniana to be less susceptible to oxygen isotopic exchange or dissolution. Each batch was cultured at 15 °C in an LMS 400 controlled temperature cabinet (LMS Ltd, Sevenoaks, UK) illuminated with a photon flux of 200 μmol/m^2^/s (approx. 43.6 W/m^2^) using in‐built fluorescent tubing on an 18:6 h day:night cycle. For growth medium, the CCAP recipe 'Diatom Medium' (DM) was used,^37^ buffered to pH 7 using tris(hydroxymelthyl)aminomethane (TRIS). Each sample represented an amalgamation of 80 L of culture, spread over eight 10‐L polycarbonate carboys and two cabinets. Each carboy was aerated by bubbling sterile filtered air, which ensured perpetual cell suspension within the culture. Periodic subsamples were collected for cell density determination using a Coulter counter (Beckman Coulter Inc., Brea, CA, USA) and a Neubauer haemocytometer. Cells were harvested by centrifugation at the onset of the stationary growth phase in order to maximise harvested biomass. Following centrifugation, the organic diatom pellet was frozen at −80 °C and freeze‐dried for storage.

Removal of organic matter was undertaken using a sequential digestion using H_2_O_2_, HNO_3_ + HCl (aqua regia; 1:3 ratio) and conc. HCl at 60 °C in a heated water bath, with repeated rinsing in 10 MΩ deionised water between phases. Due to the amount and resistance of the organic‐rich samples, the digestion procedure took 1 week to reach completion. The final mass of silica per sample was of the order of 0.5 g.

In order to assess the oxygen isotopic exchange between fresh diatoms and water, two waters of different δ^18^O_water_ values were used: deionised water (DI, 10 MΩ, δ^18^O_water_ ≈ −6.3 to −5.9 ‰) and an ^18^O‐depleted Antarctic snow water (BAS‐Lo, δ^18^O_water_ ≈ −36.3 to −35.0 ‰). Both waters were buffered to pH 5.5 with TRIS to prevent silica dissolution. Diatom silica samples were subdivided into multiple aliquots and added to the waters for storage or drying at different temperatures.

The effect of drying on the δ^18^O_silica_ values was tested at three temperatures from diatom suspensions in both DI and BAS‐Lo. Polypropylene centrifuge tubes containing 5 mL water and ~20 mg silica were dried at 80 °C in a clean drying oven, at 40 °C in a heatblock under a laminar flow hood, and by freeze‐drying (0 °C). Of these methods, freeze‐drying and 80 °C drying were predictably most effective at removing the supernatant, while drying at 40 °C took more than 1 week to complete.

The effect of storage of the diatom silica in water on δ^18^O_silica_ values was tested at five different temperatures: 80 °C and 60 °C in drying ovens, 40 °C and 20 °C in controlled temperature incubators, and 4 °C in a refrigerator. Subsamples of diatom silica were subdivided between centrifuge tubes, mixed with 10 mL of each of the waters, firmly capped and sealed with Parafilm. Samples were stored for 1 week, after which time the silica was concentrated by centrifugation, and the supernatant water was decanted before the sample was transferred to a 2‐mL centrifuge tube. The silica samples were then frozen (4 °C) and freeze‐dried.

The oxygen isotope composition of water used in each aliquot was monitored in order to account for any changes during the course of the experiment. A subsample of the initial water source was set aside ('start water', kept constant for each aliquot) and 5‐mL samples of supernatant water were collected at the end of each experiment ('end water'). The water samples were stored refrigerated in air‐tight HDPE bottles with minimal headspace and analysed within 1 month of collection. In some circumstances during storage at 80 °C, the pressure and temperature within the experimental vial led to cracking of the lid and some evaporation of water. In these cases, the δ^18^O_water_ value of the end water was higher than that of the start water by a maximum of ~5 ‰ and the value used in subsequent calculations is the mean of the two values – i.e. assuming a linear progression in both evaporative enrichment of ^18^O in water and the degree of silica‐water isotopic exchange.

### 2.2 Isotope analysis

Stable isotope analyses were conducted at the NERC Isotope Geosciences Laboratory (Nottingham, UK). The oxygen isotopic composition of waters was analysed using the equilibration method^38^ with a Sira 10 dual‐inlet isotope ratio mass spectrometer with an Isoprep 18 equilibration device (both VG, Wilmslow, UK). The oxygen isotopic ratios (^18^O/^16^O) are expressed as δ units relative to VSMOW with an analytical precision of ±0.08 ‰.

For determination of the oxygen isotope ratio of biogenic silica, the hydrous outer layer of the frustules was removed by stepwise fluorination whereby a stoichiometric deficiency of the reagent, bromine pentafluoride (BrF_5_), was used to partially react the samples before full reaction at 500 °C with an excess of BrF_5_.^22^ The oxygen liberated was converted into CO_2_ by passing over hot graphite, following the method of Clayton and Mayeda.^39^ Oxygen yields were monitored for comparison with the calculated theoretical yield. A random selection of samples was analysed in duplicate and gave a mean reproducibility of 0.3 % (1σ), which is comparable with the 0.3% reproducibility of the standard laboratory diatomite control (BFC_mod_) both within individual batches and over the longer term.^22^ The ^18^O/^16^O ratios were measured on a MAT 253 isotope ratio mass spectrometer (ThermoFinnigan, Bremen, Germany), and normalised through laboratory standards and NBS28.^22^ The data are reported in the usual δ form, as per mil (‰) deviations from VSMOW.

### 2.3 Isotope exchange mass balance equations

By conducting paired experiments using waters of two endmember δ^18^O_water_ values, it is possible to estimate the amount of affected oxygen and the associated oxygen isotope fractionation during storage. Determination of the δ^18^O_silica_ value involves the removal of molecular water and hydrous silica during prefluorination, such that only Si‐O‐Si bound silica (Q_4_ silica) is analysed. Therefore, we can assume that a *measured* sample (i.e. post‐prefluorination) consists of two units: unaltered Q_4_ silica (with a mass *A* and δ^18^O δ*A*) and altered Q_4_ silica (mass *B*, δ^18^O δ*B*). The oxygen isotope composition of the sample should reflect the isotope mass balance of these two components. For samples stored in waters of two different δ^18^O_water_ values, these mass balances are described as:

(1a+1b) $${\delta T}_{\mathrm{DI}}=\left(A\;\cdot \;\delta A\right)+\left(\left(1-A\right)\left({\delta W}_{\mathrm{DI}}+\Delta_{\mathrm{BW}}\right)\right)\;{\delta T}_{\mathrm{BAS}}=\left(A\;\cdot \;\delta A\right)+\left(\left(1-A\right)\left({\delta W}_{\mathrm{BAS}}+\Delta_{\mathrm{BW}}\right)\right)$$

where δ*T*_(*x*)_ is the δ^18^O value of the total sample and δ*W*_(*x*)_ is the δ^18^O value of the storage water. The subscript (*x*) denotes the storage water, either DI or BAS‐Lo, and Δ_BW(*x*)_ is an approximation of the fractionation factor between water and the altered silica (*B*):

(2) $$\Delta_{\mathrm{BW}\left(\mathrm{DI}\right)}=\Delta_{\mathrm{BW}\left(\mathrm{BAS}\right)}={\mathrm{\delta B}}_{\left(x\right)}-{\mathrm{\delta W}}_{\left(x\right)}$$

Solving Equations 1a and 1b simultaneously gives the following for *A*:

(3) $$A=\frac{\left({\mathrm{\delta W}}_{\mathrm{DI}}-{\mathrm{\delta W}}_{\mathrm{BAS}}\right)-\left({\mathrm{\delta T}}_{\mathrm{DI}}-{\mathrm{\delta T}}_{\mathrm{BAS}}\right)}{{\mathrm{\delta W}}_{\mathrm{DI}}-{\mathrm{\delta W}}_{\mathrm{BAS}}}$$

enabling an estimate of the mass of Q_4_‐bound oxygen affected during storage in water *x*. Equation (3) means that it is possible to calculate *A* without knowing δ*A*, assuming that δ*A* was the same in both experiments.

Solving for *A* also permits an estimate of δ*B* and Δ_BW_ for a given value of δ*A*:

(4) $$\Delta_{\mathrm{BW}\left(x\right)}=\left(\frac{{\mathrm{\delta T}}_{\left(x\right)}-\left(A\cdot \mathrm{\delta A}\right)}{1-A}\right)-{\mathrm{\delta W}}_{\left(x\right)}$$

δ*A* is known in cases where the δ^18^O value of a sample was measured prior to the experiment; however, due to limited sample availability this was not always possible. Where δ*A* was not measured, it was estimated by applying a constant offset equivalent to that observed between the measured δ*A* and δ*T* at 4 °C. Note that estimates of Δ_BW_ are extremely sensitive to the value of δ*A* used, which limits accuracy but does not affect the overall trend.

These mass balance calculations are equivalent to those used by Labeyrie and Juillet^9^ – our value of *B* approximates their value of *x* – but with an important difference. The use of stepwise fluorination means that only Q_4_ silica was analysed in this study, whereas Labeyrie and Juillet^9^ analysed the whole sample comprising Q_4_ and Q_3_ silica, following controlled isotope exchange. Therefore, Labeyrie and Juillet's *x* represents the total amount of secondary isotope exchange within the whole sample, with an assumption that all exchange takes place between water and Q_3_ silica. By contrast, our *B* is the fraction of Q_4_‐bound oxygen altered during treatment.

## 3 RESULTS

### 3.1 Effects of drying temperature and the δ^18^O_water_ value on δ^18^O_silica_ values

**Figure 1:**
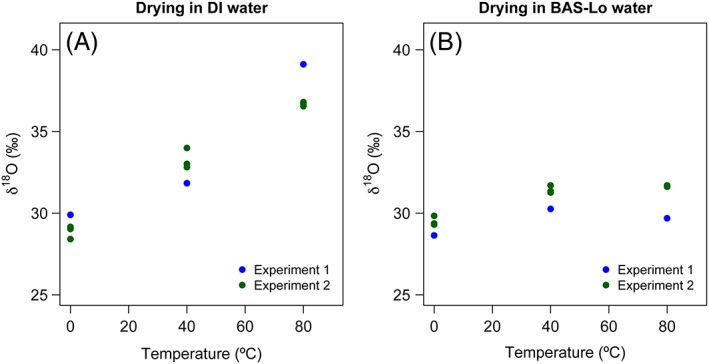
δ^18^O_silica_ values of pre‐cleaned Stephanodiscus hantzschii samples dried from two different waters at three temperatures [Color figure can be viewed at wileyonlinelibrary.com]

Two separate batches of S. hantzschii silica were used to examine the effect of drying on δ^18^O_silica_ values, with the second experiment conducted in triplicate (Table 1). Where silica samples were dried from suspension in DI, the δ^18^O_silica_ values exhibit a linear correlation with temperature (for all samples, r^2^ = 0.94, n = 12) (Figure 1A). Samples dried at 40 °C underwent a 3–5 ‰ increase in their δ^18^O_silica_ values while those dried at 80 °C underwent an average increase of 7.8 ‰ in experiment 2, and an increase of 9.2 ‰ in experiment 1 (Figure 1A). For samples stored in BAS‐Lo, the isotope change during drying was less pronounced and the difference between the two experiments greater. During experiment 1, the δ^18^O_silica_ values increased by ~1.6 ‰ during drying at 40 °C and by ~1 ‰ during drying at 80 °C (Figure 1B). In contrast, for experiment 2, the average increase in δ^18^O_silica_ values was ~1.9 ‰ at 40 °C and ~2.2 ‰ at 80 °C, but with an overlap in range at both temperatures (Figure 1B). The two experiments produced similar results, although the values for experiment 1 fall outside the range of the three values obtained in experiment 2. Such inter‐experimental differences are possibly a function of slight differences in the nature of the silica sample used. Alternatively, differences in air circulation or humidity between the two experiments may have led to differences in the evaporation of the water and isotope fractionation during evaporation, which in turn could have affected the δ^18^O_silica_ values.

**Table 1:** Raw δ^18^O_silica_ and δ^18^O values of initial water (δ*W*) data for drying experiments. Drying temperature of 0°C indicates freeze drying

Species/Experiment	Storage temp. (ºC)	Drying temp. (ºC)	Water	δW (initial)	δ^18^O_silica_ (dry)
*Stephanodiscus hantzschii* #1	20	0	*DI*	−7.1	29.9
*Stephanodiscus hantzschii* #1	20	40	*DI*	−7.1	31.8
*Stephanodiscus hantzschii* #1	20	80	*DI*	−7.1	39.1
*Stephanodiscus hantzschii* #2	4	0	*DI*	−6.3	28.4
*Stephanodiscus hantzschii* #2	4	0	*DI*	−6.3	29.0
*Stephanodiscus hantzschii* #2	4	0	*DI*	−6.3	29.2
*Stephanodiscus hantzschii* #2	4	40	*DI*	−6.3	34.0
*Stephanodiscus hantzschii* #2	4	40	*DI*	−6.3	33.0
*Stephanodiscus hantzschii* #2	4	40	*DI*	−6.2	32.8
*Stephanodiscus hantzschii* #2	4	80	*DI*	−6.4	36.7
*Stephanodiscus hantzschii* #2	4	80	*DI*	−6.2	36.5
*Stephanodiscus hantzschii* #2	4	80	*DI*	−6.4	36.8
*Stephanodiscus hantzschii* #1	20	0	*BAS‐Lo*	−36.9	28.6
*Stephanodiscus hantzschii* #1	20	40	*BAS‐Lo*	−36.9	30.3
*Stephanodiscus hantzschii* #1	20	80	*BAS‐Lo*	−36.9	29.7
*Stephanodiscus hantzschii* #2	4	0	*BAS‐Lo*	−35.3	29.8
*Stephanodiscus hantzschii* #2	4	0	*BAS‐Lo*	−35.1	29.4
*Stephanodiscus hantzschii* #2	4	0	*BAS‐Lo*	−34.9	29.3
*Stephanodiscus hantzschii* #2	4	40	*BAS‐Lo*	−35.3	31.3
*Stephanodiscus hantzschii* #2	4	40	*BAS‐Lo*	−35.0	31.7
*Stephanodiscus hantzschii* #2	4	40	*BAS‐Lo*	−35.2	31.3
*Stephanodiscus hantzschii* #2	4	80	*BAS‐Lo*	−35.0	31.7
*Stephanodiscus hantzschii* #2	4	80	*BAS‐Lo*	−35.0	31.6
*Stephanodiscus hantzschii* #2	4	80	*BAS‐Lo*	−35.0	31.7

### 3.2 Effects of storage temperature and the δ^18^O_water_ value on δ^18^O_silica_ values

**Figure 2:**
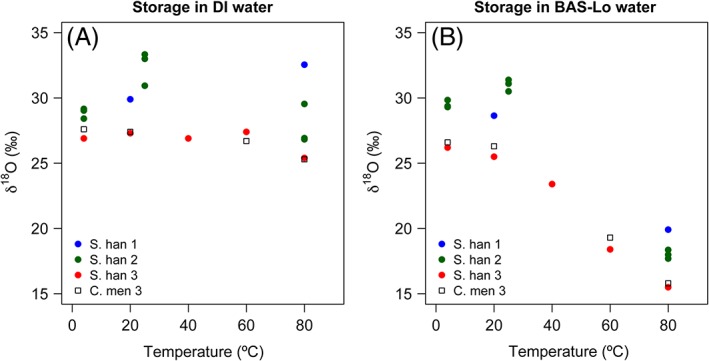
δ^18^O_silica_ values of pre‐cleaned Stephanodiscus hantzschii and Cyclotella meneghiniana samples stored for 1 week in waters of two different δ^18^O_water_ values at a range of temperatures [Color figure can be viewed at wileyonlinelibrary.com]

Four batches of diatom silica (three of S. hantzschii and one of C. meneghiniana) were used to investigate the effect of storage temperature on δ^18^O_silica_ values (Table 2). For samples stored in DI water, the relationship between the δ^18^O_silica_ values and temperature appears extremely variable when viewing the raw data and any trend is largely concealed by the range of values between experiments (Figure 2A). The most coherent pattern is evident from the results of experiment 3, namely a slight decrease in δ^18^O_silica_ values with increasing temperature, exhibited in both S. hantzschii and C. meneghiniana (Figure 2A). By contrast, data from experiment 2 suggest that the δ^18^O_silica_ value was highest at 25 °C, with a subsequent decrease at 80 °C (Figure 2A).

**Table 2:** Raw δ^18^O_silica_ and δ*W* data for storage experiments, plus calculated values for fraction of affected and unaffected oxygen (B and A, respectively), the δ^18^O_silica_ values of altered silica (δ*B*_DI_ and δ*B*_BAS_) and the isotopic fractionation factor during secondary alteration (Δ*B* − *W*). Values of δ*A* marked with * are those estimated by applying a constant –0.6‰ offset from δ^18^O_silica_ values following 1 week of storage at 4°C

Species/Experiment	Storage temp. (ºC)	δW_DI_ (start)	δW_DI_ (end)	δW_DI_ (mean)	δW_BAS_ (start)	δW_BAS_ (end)	δW_BAS_ (mean)	δA	δ^18^O_silica_ (DI)	δ^18^O_silica_ (BAS)	A	B	δB_DI_	δB_BAS_	ΔB‐W
*Stephanodiscus hantzschii* #1	25.0			−7.1			−36.9	N/A	29.9	28.6	0.96	0.04			
*Stephanodiscus hantzschii* #1	80.0			−6.9			−36.5	N/A	32.5	19.9	0.57	0.43			
*Stephanodiscus hantzschii* #2	4.0	−6.3	−6.3	−6.3	−35.0	−35.3	−35.2		29.8	28.4					
*Stephanodiscus hantzschii* #2	4.0	−6.3	−6.3	−6.3	−35.0	−35.1	−35.1		29.4	29.0					
*Stephanodiscus hantzschii* #2	4.0	−6.3	−6.3	−6.3	−35.0	−34.9	−35.0		29.3	29.2					
Mean	4.0			−6.3			−35.1	28.9*	29.5	28.9	0.98	0.02	56.4	27.6	62.7
*Stephanodiscus hantzschii* #2	25.0	−6.3	−6.3	−6.3	−35.0	−34.9	−35.0		30.9	30.5					
*Stephanodiscus hantzschii* #2	25.0	−6.3	−6.3	−6.3	−35.0	−34.8	−34.9		33.3	31.1					
*Stephanodiscus hantzschii* #2	25.0	−6.3	−6.2	−6.3	−35.0	−35.0	−35.0		33.0	31.4					
Mean	25.0			−6.3			−35.0	28.9*	32.4	31.0	0.95	0.05	99.6	70.9	105.9
*Stephanodiscus hantzschii* #2	80.0	−6.3	−1.2	−3.7	−35.0	−29.5	−32.3		29.5	17.7					
*Stephanodiscus hantzschii* #2	80.0	−6.3	−4.2	−5.3	−35.0	−33.9	−34.5		26.9	18.0					
*Stephanodiscus hantzschii* #2	80.0	−6.3	−5.6	−6.0	−35.0	−33.8	−34.4		26.8	18.4					
Mean	80.0			−5.0			−33.7	28.9*	27.8	18.0	0.66	0.34	25.6	−3.2	30.5
*Stephanodiscus hantzschii* #3	4.0	−5.9	−6.0	−5.9	−36.3	−36.2	−36.2	26.3	26.9	26.2	0.97	0.03	51.6	21.3	57.5
*Stephanodiscus hantzschii* #3	20.0	−5.9	−5.9	−5.9	−36.3	−36.3	−36.3	26.3	27.3	25.5	0.94	0.06	43.0	12.7	48.9
*Stephanodiscus hantzschii* #3	40.0	−5.9	−5.9	−5.9	−36.3	−36.3	−36.3	26.3	26.9	23.4	0.88	0.12	31.4	1.0	37.3
*Stephanodiscus hantzschii* #3	60.0	−5.9	−5.7	−5.8	−36.3	−36.5	−36.4	26.3	27.4	18.4	0.71	0.29	30.1	−0.5	35.9
*Stephanodiscus hantzschii* #3	80.0	−5.9	−4.7	−5.3	−36.3	−35.2	−35.7	26.3	25.4	15.5	0.67	0.33	23.6	−6.8	28.9
*Cyclotella meneghiniana #1*	4.0	−5.9	−5.9	−5.9	−36.3	−36.4	−36.3	27.0	27.6	26.6	0.97	0.03	45.3	14.9	51.2
*Cyclotella meneghiniana #1*	20.0	−5.9	−6.0	−5.9	−36.3	−36.4	−36.3	27.0	27.4	26.3	0.96	0.04	36.9	6.5	42.9
*Cyclotella meneghiniana #1*	60.0	−5.9	−5.8	−5.9	−36.3	−36.0	−36.1	27.0	26.7	19.3	0.75	0.25	25.9	−4.3	31.8
*Cyclotella meneghiniana #1*	80.0	−5.9	−4.6	−5.3	−36.3	−35.2	−35.7	27.0	25.3	15.8	0.69	0.31	21.5	−8.9	26.8

For samples stored in BAS‐Lo water, the relationship between δ^18^O_silica_ values and temperature is much more consistent between experiments, accentuated by a greater magnitude of change. As is the case with storage in DI water, there appears to be a slight increase in δ^18^O_silica_ values for samples stored at 25 °C during experiments 1 and 2; however, the overall pattern is a negative correlation between temperature and δ^18^O_silica_ values whereby samples stored at 80 °C exhibited a ~12 ‰ decrease in their δ^18^O_silica_ values relative to those stored at 4 °C (Figure 2B). Experiment 3 involved samples of both S. hantzschii and C. meneghiniana, and the pattern of change in the δ^18^O_silica_ values in response to temperature is near identical for both taxa (Figure 2), particularly where samples were stored in BAS‐Lo water (Figure 2B).

### 3.3 Effect of temperature on the mass alteration of silica‐bound oxygen

**Figure 3:**
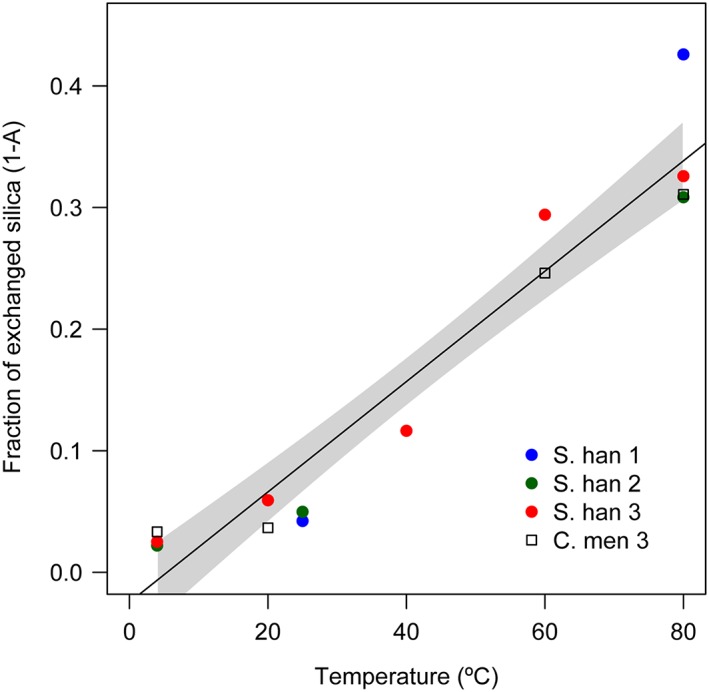
Fraction of affected silica‐bound oxygen vs storage temperature, following storage for 1 week in water. Values calculated using Equation (3), where *B* is equal to the fraction of affected oxygen. Solid black line = linear regression fit, grey shaded area = 95% confidence intervals [Color figure can be viewed at wileyonlinelibrary.com]

The fractional mass of altered silica‐bound oxygen during storage was estimated using Equation (4), for which all parameters were measured. The estimated fraction of affected oxygen is linearly correlated with temperature (r^2^ = 0.93, n = 20), ranging from ~0 at 4 °C to ~0.3–0.4 at 80 °C (Figure 3).

### 3.4 Effect of temperature on isotope fractionation during secondary alteration of oxygen isotopes in diatom silica

**Figure 4:**
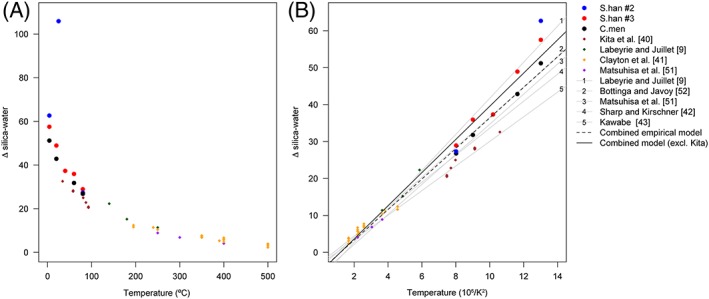
Summary of published (diamonds) and new data (circles) for silica‐water oxygen isotope fractionation, and extrapolated fractionation models based on experiments using quartz, amorphous silica and secondary exchange with diatom silica. Plotted against temperature in °C (A), and 10^6^/*T* ^2^ (in Kelvin; b). In (B), S. han #2 data for 25 °C have been omitted [Color figure can be viewed at wileyonlinelibrary.com]

The oxygen isotope fractionation (Δ_BW_) between altered Q_4_ silica (*B*) and water was estimated using Equation (4). When expressed as a fractionation, much of the scatter in Figure 2A is diminished, suggesting that a large proportion of the between‐sample differences can be explained as a function of variations in δ*W* and in the amount of oxygen affected (Figure 4A). In this case, an added source of uncertainty is the oxygen isotopic composition of the initial Q_4_ silica (δ*A*). Here, δ*A* is assumed to be equivalent to the oxygen isotopic composition of the total sample at the beginning of the experiment, whereby prefluorination removes all non‐Q_4_ silica.^22^ However, δ*A* was measured only for the *Stephanodiscus* #3 experiment, where δ*A* differed from the post‐treatment δ^18^O_silica_ value at 4 °C (δ*T*_4°C_) by 0.6 ‰. Consequently, where measured values for δ*A* were absent, a correction of −0.6‰ was applied to the δ*T*_4°C_ values to estimate δ*A*. Application of this correction to all samples defines a temperature‐fractionation relationship that is consistent with that defined by the data from *Stephanodiscus* experiment #3 (Figure 4), except for a single sample, *Stephanodiscus #*2, 25 °C, which is a clear outlier (Figure 4A). Irrespective of this outlier, there is a negative correlation between Δ_BW_ and temperature (Figure 4), a relationship which appears to conform with the low‐temperature extrapolation of published high‐temperature silica‐water oxygen isotope fractionation models (Figures 4A and 4B). These published models were established through a range of experiments, including secondary alteration of diatom silica^9^ and precipitation of amorphous silica^40^ and quartz.^41^, ^42^, ^43^

## 4 DISCUSSION

The results presented here support previous evidence for instability in the δ^18^O values of freshly synthesised biogenic silica, which continues to undergo oxygen isotope fractionation post‐mortem. Previous studies have reported similar patterns from diatom silica collected in the natural environment^10^, ^13^, ^27^ and under experimental conditions.^14^ The mechanisms behind post‐mortem modification of δ^18^O_silica_ values may relate to a number of individual processes or combinations thereof. These include methodological issues (incomplete removal of Q_3_ silica; contamination by mineral silica or organic matter) or naturally occurring processes (dissolution; secondary silicate precipitation; condensation of Q_3_ to Q_4_ silica). In this circumstance, we discount methodological artefacts with the following justification. First, the stepwise fluorination method is designed to react away loosely bonded hydration water and silanol prior to the liberation of Q_4_‐bound oxygen at 500 °C.^22^ In principle, it is possible that some Q_3_ silica survives this process, which would lead to oxygen isotope contamination by formerly exchangeable silica; however, this is deemed unlikely given the high reactivity of silanol with BrF_5_. Furthermore, inter‐laboratory comparisons with other methods which do not involve pre‐fluorination indicate that our method is robust, including during analysis of silanol‐rich silica standards.^26^ Retention of Q_3_ silica post‐prefluorination should also lead to a consistent negative bias in δ^18^O_silica_ values,^13^, ^44^ which is not observed in our data (Figures 1 and 2). Secondly, contamination is discounted on the basis that the diatom culture material was never in contact with mineral silica and a prolonged oxidation stage was carried out in excess of usual requirements for removal of organic constituents.^45^ Even in the case of some organic matter being present, this would be constant between samples for a particular experiment, and would therefore not account for the differences between treatments. The consistency between experiments, including between different diatom taxa, also attests to the likelihood of differences in organic contamination having a negligible effect on our results.

With respect to naturally occurring processes, dissolution is a common feature of diatom silica diagenesis^46^ and the dissolution effect on δ^18^O_silica_ values has not been well characterised to date. Experimental studies have indicated that diatom silica is isotopically homogenous and that δ^18^O_silica_ values are not susceptible to dissolution effects.^10^, ^31^ Similar conclusions were drawn by Moschen et al,^27^ although they did observe an increase in δ^18^O_silica_ values under high pH conditions. In our experiments, dissolution was mitigated by buffering solutions at pH 5.5, and we suggest that dissolution is unlikely to have accounted for the observed variability in δ^18^O_silica_ values. In the case of some dissolution, it is reasonable to expect that this will preferentially affect the lighter isotope (^16^O), leading to an increase in the δ^18^O value of the residual particulate silica,^27^, ^47^ a pattern which is not consistently observed in our data (Figures 1 and 2). In addition, although our comparison between diatom taxa was by no means exhaustive, the absence of any significant difference between data for S. hantzschii and C. meneghiniana suggests that whatever causes the secondary alteration in δ^18^O_silica_ values overrides any effect of the frustule's surface area/volume or silica density. This in turn suggests that dissolution was not the principal cause of the change in δ^18^O_silica_ values, since we expect dissolution to affect the less dense S. hantzschii to a greater extent than C. meneghiniana.

Secondary precipitation of silica or isotope exchange between silica and water coupled with silica condensation are two additional processes which may result in post‐mortem changes in δ^18^O_silica_ values. Secondary precipitation can occur via silicate coating of diatom frustules in the presence of Al^48^ or protein‐catalysed precipitation of particulate silica.^13^, ^49^ To date, neither process has been demonstrated to contribute to post‐mortem δ^18^O_silica_ modification^13^ but both would require saturating concentrations of dissolved silica, hence a degree of coupling with prior silica dissolution. By contrast, there is clear evidence to indicate post‐mortem δ^18^O_silica_ alteration as a function of silica condensation.^10^, ^14^, ^27^ First, the condensation of hydrated Q_3_ silica to Q_4_ appears to be a common process during both laboratory treatment and natural sedimentary diagenesis. A review of published nuclear magnetic resonance (NMR) measurements of the Q_4_/Q_3_ ratios of biogenic silica indicates a range of values which vary as a function of prior chemical treatment and depositional age.^20^ For example, freshly cultured, untreated marine diatoms had a Q_4_/Q_3_ range between 1.5 and 2^17^, ^18^, ^50^ while diatom frustules treated by boiling in 2% SDS and 0.1 M EDTA had Q_4_/Q_3_ ratios of 2.5–2.8.^17^, ^50^ By contrast, Miocene fossil diatoms had a Q_4_/Q_3_ ratio of 6.1,^18^ whereas two diatom standards – fossil diatoms from the Southern Ocean (PS) and continental Africa (BFC) – have Q_4_/Q_3_ ratios of 2.2 and 3.1, respectively.^26^ These measurements imply that heating in the laboratory or natural aging leads to a progressive increase in Q_4_/Q_3_. Such structural changes have also been observed through Fourier transform infrared (FTIR) and surface charge density analysis of sedimenting and experimentally altered diatoms, in accordance with post‐mortem changes in δ^18^O_silica_ values.^10^, ^14^, ^27^ Silica condensation, whereby silanols combine in the construction of new Si‐O‐Si bonded silica, with the loss of water, can be described by the following reaction:

(5) $$\mathrm{Si}{\left(O-\mathrm{Si}\right)}_{3}\mathrm{OH}+\mathrm{Si}{\left(O-\mathrm{Si}\right)}_{3}\mathrm{OH}\to \mathrm{Si}{\left(O-\mathrm{Si}\right)}_{4}+\mathrm{Si}{\left(O-\mathrm{Si}\right)}_{4}+H_{2}O$$

Importantly, during this condensation reaction, one of the silanol‐bound oxygen atoms becomes fixed within a new Si‐O‐Si matrix, thus entraining previously exchangeable oxygen.^9^, ^23^ Silica condensation following isotope exchange between Q_3_ silica and water is therefore an established process with a physical basis that can explain post‐mortem δ^18^O_silica_ modification, although further research is required in order to categorically establish the rate of structural changes that lead to post‐mortem alteration of δ^18^O_silica_ values.

### 4.1 Temperature effects over post‐mortem changes in δ^18^O_silica_ values

Despite limitations in our understanding of the processes behind post‐mortem isotope alteration, estimating the relative mass of oxygen affected and the associated isotope fractionation can provide a useful insight into the potential mechanisms and their impact on δ^18^O_silica_‐based research. Such estimates were achieved by conducting paired experiments and solving simultaneous mass balance equations (Equations 1–4). Following this approach, the fraction of Q_4_‐bound oxygen affected during each treatment was positively correlated with temperature (Figure 3; r^2^ = 0.92), a relationship similar to previous observations of the exchange between Q_3_ silica and water vapour.^9^ The extent to which this response can be extrapolated beyond the measured range (4–80 °C) is uncertain. In particular, we have no reason to expect that the magnitude of secondary exchange would continue to decline linearly at temperatures below the measured range (i.e. <4 °C). Similarly, the maximum potential for post‐mortem alteration remains a matter of debate, either with increasing temperature or for prolonged periods of time. Dodd et al^13^, ^14^ argue that the δ^18^O_silica_ values of diatoms in both experimental and natural settings undergo alteration along a continuum towards complete re‐equilibration with ambient conditions in line with inorganic fractionation. However, other studies have suggested that siloxane is isotopically stable, and thus the maximum extent of secondary isotope alteration is limited by the initial mass of exchangeable silanol,^1^, ^9^, ^23^ which would in effect rule out the potential for complete re‐equilibration within the sedimentary environment. This second hypothesis is consistent with the interpretation that post‐mortem δ^18^O_silica_ changes originate from condensation of Q_3_ to Q_4_ silica, with the initial mass of Q_3_ silica acting as the limiting factor for secondary alteration.

The similarity between our estimated temperature‐dependent oxygen isotope fractionation during post‐mortem δ^18^O_silica_ change and previous calculations based on high temperature silica‐water experiments (Figure 4) suggests that the incorporation of a secondary isotope signature in diatoms has analogues in inorganic silica precipitation.^9^, ^13^, ^40^, ^41^, ^42^, ^51^, ^52^, ^53^ For the purpose of subsequent discussion, we combined our data with published empirical values for temperatures <250 °C to fit a linear regression. This was performed with and without the data of Kita et al,^40^ which plot consistently 3–5 ‰ lower than our data and thus influence both the strength and the slope of the temperature vs fractionation model (Figure 4). The combined datasets define the following models: Δ_BW_ = 4.14(10^6^ *T* ^−2^) − 4.96, r^2^ = 0.94 (with data from Kita et al included), and Δ_BW_ = 4.49(10^6^ *T* ^−2^) − 5.34, r^2^ = 0.98 (excluding the data from Kita et al).

### 4.2 Implications of secondary isotope exchange for laboratory protocols

A major consequence of the findings reported here relates to the treatment of biogenic silica during preparation for isotope analysis. In the acquisition of δ^18^O_silica_ data, it is a necessary prerequisite to obtain pure biogenic silica samples, free of organic and mineralogical contamination. Consequently, sample preparation invariably involves prolonged heating in aqueous oxidising reagents, in addition to physical treatments such as wet sieving, settling, micromanipulation or heavy liquid separation.^1^, ^29^, ^54^, ^55^ Particularly striking is the influence of drying on δ^18^O_silica_ values (Figure 1). This response can be explained by the combined effects of an evaporation‐driven increase in δ^18^O_water_ values and the acceleration of oxygen exchange under heating and drying. Evaporation leads to a progressive increase in δ^18^O_water_, until the point of total evaporation. Consequently, if higher temperatures lead to an increased magnitude of oxygen exchange, the ^18^O‐enriched water will translate as an increase in δ^18^O_silica_ values. Interestingly, the δ^18^O_silica_ values of samples dried from suspension in BAS‐Lo water exhibit a weaker relationship with temperature. We suggest this is because the low δ^18^O_water_ value of BAS‐Lo water compensates for the evaporative ^18^O enrichment, resulting in a net effect of negligible change (Figure 1B). It has been commonplace for some researchers to dry both sediments and purified silica samples in conventional drying cabinets at high temperatures. Our findings demonstrate that caution is required when both drying and storing biogenic silica in aqueous reagents as this can lead to marked changes in δ^18^O_silica_ values, particularly at high temperatures.

The stability of δ^18^O_silica_ values under heating appears to vary according to the nature of the samples and the duration of contact with water. For example, Tyler et al^45^ tested a number of organic removal treatments and found that heating at 70 °C in H_2_O_2_ for 2 weeks, and heating to 550 °C in a furnace, resulted in negligible alteration to the δ^18^O_silica_ value of a Miocene diatomite sample. In this example, the absence of any change in the δ^18^O_silica_ value may reflect the sample age or crystallography, with a low fraction of exchangeable oxygen post silica maturation. In a similar experiment, Crespin et al^24^ examined the effect of digestion temperature on δ^18^O_silica_ values and suggested that 60 °C is an optimal temperature, below which organic contaminants were not effectively digested, and above which δ^18^O_silica_ changes were observed.

**Figure 5:**
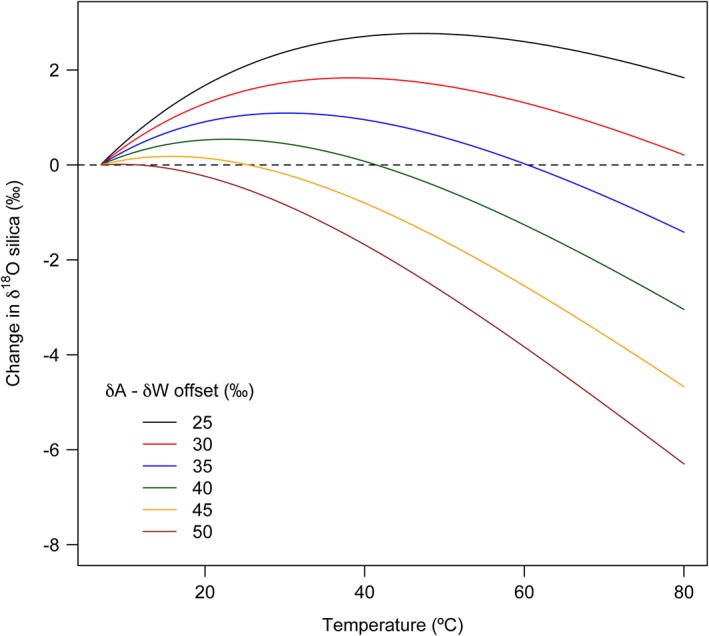
Hypothetical modification of δ^18^O_silica_ values with temperature as a combined function of mass exchange (Figure 3) and silica‐water oxygen isotope fractionation (Figure 4; combined model excluding data from Kita et al^40^). Offsets represent the difference between the isotopic composition of the initial sample and the host water/reagent. Modelled values are representative of cultured freshwater diatoms such as those reported, and different samples may vary in their propensity for isotope exchange [Color figure can be viewed at wileyonlinelibrary.com]

The empirical calibrations defined here suggest that the stability of δ^18^O_silica_ values during treatment can be explained by a combination of the oxygen isotopic composition of reagent and initial silica, the silica condensation rate and the temperature dependency over Δ_BW_. For example, Figure 5 illustrates the potential isotopic alteration with temperature for six hypothetical sample‐reagent combinations based on our empirical calibrations (Equations 1–4). If the net modification to the δ^18^O_silica_ value is a combined function of increasing oxygen exchange with temperature (Figure 3) and decreasing silica‐water oxygen isotope fractionation (Figure 4), the δ^18^O_silica_ value of a particular sample can either decrease or increase with temperature, depending on the difference in the δ^18^O value between the initial sample (δ*A*) and the reagent (δ*W*). At one extreme, where δ*A* − δ*W* = 50 ‰ (e.g. a silica sample of 40 ‰ is placed in a reagent of −10‰), the δ^18^O_silica_ value will decrease with increasing temperature as the silica obtains a new signal from the host water. However, where δ*A* − δ*W* is only 25 ‰ (e.g. a silica sample of 20 ‰ and a reagent of −5‰), heating in an aqueous reagent could lead to modified δ^18^O_silica_ values less than, greater than or equal to that of the initial sample (Figure 5). Dodd and Sharp^44^ also experimented with sample digestion protocols, by digesting biogenic silica in isotopically labelled HNO_3_ for 12 h at 90 °C. Whilst they observed a clear alteration to the δ^18^O value of Q_3_ silica within the initial phase of stepwise fluorination, the treatment did not affect the δ^18^O_silica_ value of Q_4_. It is possible that the 12‐h digestion used by Dodd and Sharp^44^ was sufficiently brief to avoid significant oxygen isotopic modification, or that the sample analysed contained a high percentage of isotopically stable Q_4_ silica. Following these observations, it is clear that future methodological protocols should be adapted to circumvent potential oxygen isotope alteration during laboratory treatment. The obvious solution would be to find a means of effectively removing organic detritus without recourse to excessive heating or prolonged treatment. In this respect, the use of perchloric acid, as proposed by Crespin et al,^24^ may provide a successful solution. In addition, the use of organic solvents and detergents may facilitate preliminary denaturing of organic compounds prior to conventional oxidation techniques. In the short term, sample preparation techniques require optimisation and validation according to the nature of the sample in hand.

### 4.3 Implications of post‐mortem isotope alteration for palaeoclimate research

**Figure 6:**
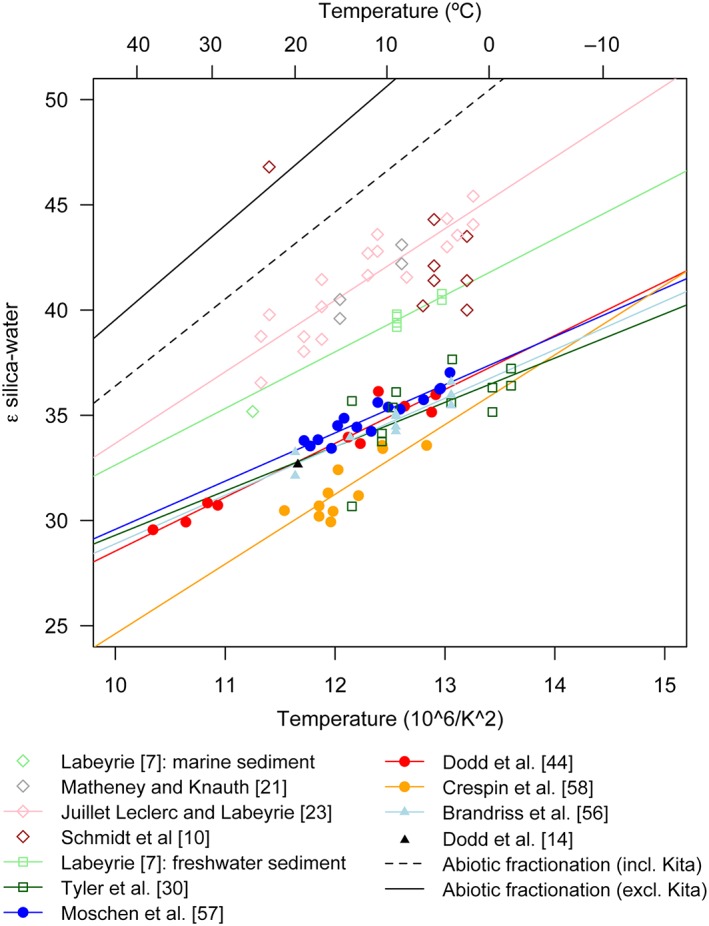
Summary of published silica‐water oxygen isotope fractionation calibrations for diatom silica vs temperature. Open symbols represent surface sediment‐based calibrations: open diamonds are marine sediments; open squares are lake sediments; closed circles represent living or sedimenting diatoms sampled from freshwaters; closed triangles represent cultured freshwater diatoms. The solid and dashed black lines are abiotic silica‐water oxygen isotope fractionation models defined in Figure 4 [Color figure can be viewed at wileyonlinelibrary.com]

In addition to the methodological challenge presented by post‐mortem isotope alteration, it is also likely that similar processes influence the δ^18^O_silica_ values of sedimentary deposits in the natural environment. A number of studies have reported marked differences in the δ^18^O_silica_ values of living vs sedimentary diatoms in freshwater and marine environments,^10^, ^13^, ^27^ and it is possible that those observations and our experimental results relate to common mechanisms. With respect to the controls over δ^18^O_silica_ values in sediment records, an emerging consensus suggests that oxygen isotope fractionation within living diatoms is subject to a temperature dependency of ~ −0.2 ‰/°C, as measured from freshwater diatoms in culture^56^ and modern lake communities^44^, ^57^, ^58^ (Figure 6). The initial δ^18^O_silica_ values and silica‐water fractionation factor measured in our experiments also appear to conform with the above‐mentioned studies, though we add the caveat that our experiments were not optimised to precisely constrain the silica‐water oxygen isotope fractionation factor due to the large volumes of diatoms cultured and the intensive oxidation of organic matter which was necessary prior to analysis. In contrast, post‐mortem isotope fractionation appears to exhibit a temperature dependency of ~ −0.36 ‰/°C (in the range 0–40 °C), analogous to equilibrium silica‐water oxygen isotope fractionation at high temperatures^9^, ^14^, ^41^, ^42^, ^51^, ^52^, ^53^ (Figure 4). Sedimentary diatom silica values usually fall between these endmembers, suggesting either partial or complete oxygen isotope re‐equilibration within sediments^13^, ^14^, ^44^ (Figure 6). Such re‐equilibration will lead to higher δ^18^O_silica_ values within sediments than in living diatoms, and a smoothing of seasonal patterns towards multiannual average conditions; however, further research is required in order to fully describe the rate and extent of secondary silica condensation and isotopic re‐equilibration in natural and experimental settings.

## 5 CONCLUSIONS

The effects of post‐mortem oxygen isotope fractionation on the δ^18^O values of diatom silica are demonstrated and quantified through experiments with cultured freshwater diatoms. Heating and drying diatom silica results in significant alteration of the original δ^18^O_silica_ signature, with implications for laboratory protocols and interpretation of palaeoclimate records. Although the mechanisms remain uncertain, it is suggested that this secondary alteration may be related to silica condensation, associated with the loss of hydroxyl silica and water in the formation of new Si‐O‐Si bonded silica. In the laboratory, the amount of oxygen affected during storage is positively correlated with temperature. The associated isotope fractionation conforms to the range of extrapolated models based on high‐temperature inorganic silica‐water oxygen isotope calibrations. The range of δ^18^O_silica_ values observed in our experiments is compatible with observed offsets between the δ^18^O_silica_ values of fresh and sedimentary diatoms in both marine and freshwater environments. Therefore, consideration of such effects is critical both in the laboratory handling of fresh diatom silica and in the interpretation of δ^18^O_silica_ values as a palaeoclimate tracer.
